# Neural stem cell sparing by linac based intensity modulated stereotactic radiotherapy in intracranial tumors

**DOI:** 10.1186/1748-717X-8-187

**Published:** 2013-07-24

**Authors:** Julia Oehler, Tim Brachwitz, Thomas G Wendt, Nico Banz, Mario Walther, Tilo Wiezorek

**Affiliations:** 1 Department of Radiation Oncology, Jena University Hospital, Friedrich-Schiller-University Jena, Bachstrasse 18, Jena, D-07743, Germany; 2 Department of Radiation Oncology, Division of Medical Physics, Jena University Hospital, Friedrich-Schiller-University Jena, Bachstrasse 18, Jena, D-07743, Germany; 3 Institute of Medical Statistics, Computer Sciences and Documentation, Jena University Hospital, Friedrich-Schiller-University Jena, Bachstrasse 18, Jena, D-07743, Germany

**Keywords:** Stereotactic radiotherapy, Intensity modulated radiotherapy, Brain tumor, Intracranial tumor, Neurocognitive decline, Hippocampus, Subventricular zone, Neural stem cell

## Abstract

**Background:**

Neurocognitive decline observed after radiotherapy (RT) for brain tumors in long time survivors is attributed to radiation exposure of the hippocampus and the subventricular zone (SVZ). The potential of sparing capabilities for both structures by optimized intensity modulated stereotactic radiotherapy (IMSRT) is investigated.

**Methods:**

Brain tumors were irradiated by stereotactic 3D conformal RT or IMSRT using m3 collimator optimized for PTV and for sparing of the conventional OARs (lens, retina, optic nerve, chiasm, cochlea, brain stem and the medulla oblongata). Retrospectively both hippocampi and SVZ were added to the list of OAR and their dose volume histograms were compared to those from two newly generated IMSRT plans using 7 or 14 beamlets (IMSRT-7, IMSRT-14) dedicated for optimized additional sparing of these structures. Conventional OAR constraints were kept constant. Impact of plan complexity and planning target volume (PTV) topography on sparing of both hippocampi and SVZ, conformity index (CI), the homogeneity index (HI) and quality of coverage (QoC) were analyzed. Limits of agreement were used to compare sparing of stem cell niches with either IMSRT-7 or IMSRT-14. The influence of treatment technique related to the topography ratio between PTV and OARs, realized in group A-D, was assessed by a mixed model.

**Results:**

In 47 patients CI (p ≤ 0.003) and HI (p < 0.001) improved by IMSRT-7, IMSRT-14, QoC remained stable (p ≥ 0.50) indicating no compromise in radiotherapy. 90% of normal brain was exposed to a significantly higher dose using IMSRT. IMSRT-7 plans resulted in significantly lower biologically effective doses at all four neural stem cell structures, while contralateral neural stem cells are better spared compared to ipsilateral. A further increase of the number of beamlets (IMSRT-14) did not improve sparing significantly, so IMSRT-7 and IMSRT-14 can be used interchangeable. Patients with tumors contacting neither the subventricular zone nor the cortex benefit most from IMSRT (p < 0.001).

**Conclusion:**

The feasibility of neural stem cell niches sparing with sophisticated linac based inverse IMSRT with 7 beamlets in an unselected cohort of intracranial tumors in relation to topographic situation has been demonstrated. Clinical relevance testing neurotoxicity remains to be demonstrated.

## Introduction

Research in the field of neural stem cells has made a quantum leap and yielded complete new insights upon the regeneration of brain cells and functions during the last 15 years. The hippocampal precursor cells, that generate new neurons with their particular function, represent a ‘neurogenic reserve’ – the potential to remain flexible and plastic in hippocampal learning [1]. Neural stem cells reside in the subventricular zone (SVZ) of the adult mammalian brain. It is concluded that SVZ astrocyte like cells act as neural stem cells in both the normal and regenerating brain [2-4]. The dentate granule cell layer of the hippocampal formation has the distinctive property of ongoing neurogenesis, that continues throughout adult life. Although the function of these newly generated neurons and the mechanisms that control their birth are not yet fully understood. Age, activity and psychosocial stress have all been demonstrated to regulate this type of neurogenesis. Radiation- and chemotherapy-induced damage to progenitor populations, responsible for maintenance of white matter integrity and adult hippocampal neurogenesis, is now believed to play a major role in the neurocognitive impairment many cancer survivors experience [5,6]. Therefore functional recovery paved to a major part by cellular restoring may be compromised by radiation therapy.

We explored the potential of modern intensity modulated stereotactic radiotherapy (IMSRT) for sparing the hippocampus, harboring the dentate gyrus and the subventricular zones (SVZ) adjacent to the lateral ventricles.

## Material and methods

All consecutively treated patients, who received high dose fractionated stereotactic radiotherapy for intracranial tumors between 3/2007 and 5/2010, were retrieved from the institutional data base for analysis in this retrospective planning study. Patients with a maximum diameter of the planning target volume (PTV) of 100 mm and above were excluded. In all patients the tumor to be treated has been confirmed by histology after biopsy, partial or gross tumor resection. The histologic type of tumor is given in Table 1. Anaplastic glioma, glioblastoma (GBM) and gliosarcoma have been conventionally fractionated with 60 Gy /2 Gy (total dose/dose per fraction), low grade glioma with 54 Gy /2 Gy, all other entities with 50.4 to 54 Gy /1.8 Gy (ICRU 50). Patients with unconventional fractionation schedules were excluded. Patients with GBM and anaplastic glioma WHO °3 or °4 were given temozolomide, 75 mg/sqm body surface area, orally every treatment day. The technique applied was selected in order to match the conventional dose constraints as good as possible. Clinically applied treatment plans were generated by the following procedures and planning constraints.

**Table 1:** Demographic and tumor characteristics and radiotherapy planning details

n (male: female)	47 (26:21)
median age (range)	49.68 years (2–88)
histology	
meningioma	14
anaplastic astrocytoma	11
glioblastoma	8
oligodendroglioma	5
craniopharyngeoma	4
oligoastrocytoma	1
xantoastrocytoma	2
gliosarcoma	1
rhabdomyoid tumor	1
3 D conformal plan ^a^	27
micromultileaf collimator (m3) IMSRT ^a^	20
total radiation dose (ICRU 50)	
mean ± SD (range)	55.19 ±3.77 (50–60) Gy
PTV volume	
mean ± SD (range)	79.13 ±66.62 (2.1-255.3) ml
topographic groups ^b^ (n)	
A	11
B	8
C	14
D	14

^a^: to fulfil of conventionally defined organs at risk constraints (see text).

^b^: according to the definition of [10].

### Conventional planning procedure

For planning purposes all patients had a plain axial computed tomography (CT) with 2.5 mm slice thickness, covering the entire brain from the vertex to cervical vertebra 2. The CT data set was taken on a scanner dedicated for radiotherapy planning. The patient was fixed in the high precision mask system, encaged in the stereotactic CT localizer (Brainlab ®, Feldkirchen/Munich, Germany). A 1.5 Tesla magnet resonance image (MRI) rendering volume scan based on 192 sagittal slices with 0.9 mm thickness was acquired immediately after contrast injection for all patients to enhance contouring. For delineation, a gadolinium-enhanced T1-weighted gradient-echo sequence was used to delineate gross tumor volume (GTV), clinical target volume (CTV) and planning target volume (PTV). CT and MRI data sets were fused using the integrated fusion algorithm of the planning software iPlan RT ® version 3.0 and 4.0 (Brainlab, Feldkirchen/Munich, Germany).

GTV was contoured according to the contrast enhanced volumes. The CTV was generated by contouring a margin around the surgical defect (tumor bed) accounting for microscopic extension in case of incomplete surgical removal or biopsy only. The margin around GTV to create CTV was chosen 10 mm in high grade tumors (WHO °3 and 4) and 5–7 mm unless the scull would have been exposed in low grade tumors (WHO °2). For meningioma WHO grade 1 and craniopharyngioma, the selected margin was 2–3 mm. PTV was generated by an additional margin of 2 mm in all directions, irrespective of tumor grading to compensate for daily set-up error.

The following structures (conventional organs at risk, OARs) were routinely delineated with their maximum dose accepted in the final plan (constraints given in brackets): lens (10 Gy), retina (35 Gy), optic nerves (45–50 Gy), chiasm (45 Gy), cochlea (45 Gy), brain stem (50 Gy) and medulla oblongata (50 Gy). Contouring was performed in axial reconstructions of the MRI data set. All plans were calculated and optimized in Brainlab iPlan RT ® version 3.0 and 4.0 and applied to the patient using the micromultileaf collimator m3 (Brainlab, Feldkirchen/Munich, Germany). The preferred treatment plan used forward planned 3D conformal non-coplanar isocentric fixed beams. If 3D conformal RT planning failed to meet any of these constraints, IMSRT using the step-and-shoot technology was used. An experienced medical physicist and a radiation oncologist decided on the applied technology on an individual basis. All patients were irradiated with plans optimized for sparing of delineated OARs and yielding acceptable dose homogeneity at the PTV. The ICRU 50 criteria with regard to PTV have been fulfilled in all plans. These plans have been denominated as “not optimized” to describe the results. The normal tissue maximum doses have been recorded from the dose volume histograms (DVH) generated for all three plans in each patient.

### Planning optimized for hippocampal and subventricular zone (SVZ) sparing

**Figure 1:**
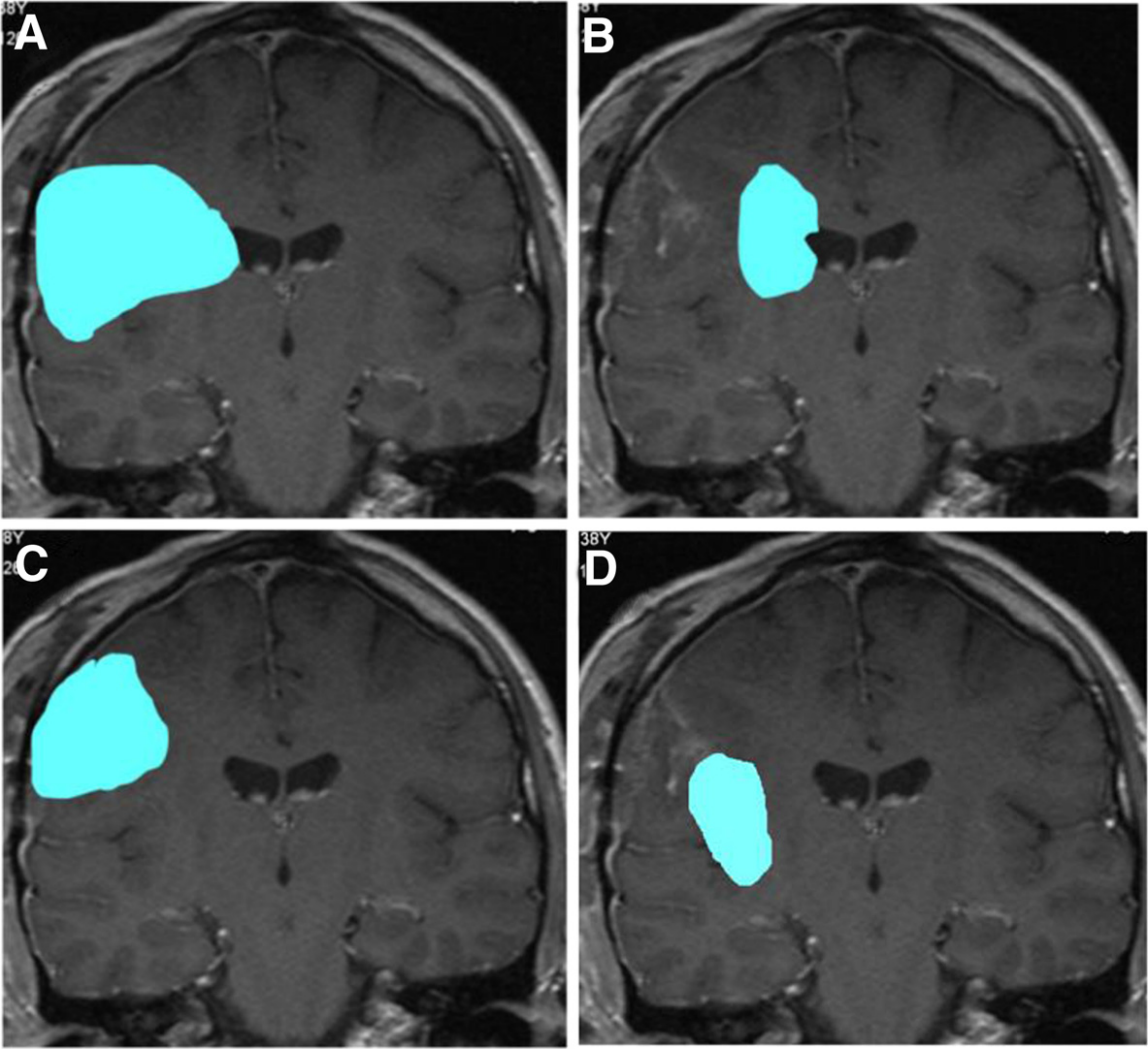
**Right temporal planning target volume (PTV, red) and organs at risk delineated in the MRI rendering volume data set.** Lenses in pale blue and purple, nn. optici and chiasm in yellow, brain stem and medulla oblongata in green, the hippocampi and the subventricular zones (SVZ) in different shades of magenta.

Neural stem cell niches were delineated in all restored original MRI or CT data sets (Figure 1) for the prospective optimization process. Left and right hippocampi were contoured with the assistance of an experienced neuroradiologist, according to published guidelines [7]. The left/right SVZ were contoured around left/right lateral and third ventricles, creating a 3–5 mm rim immediately adjacent to their lateral borders [8]. The total volume of the brain from the vertex down to the foramen magnum was calculated after the PTV has been excluded, using Boolean operators. Thus the mean dose applied to 10% (D 10), 50% (D 50) and 90% (D 90) of the normal brain volume was generated for all planning procedures in all patients.

Two intensity modulated plans were generated and optimized for the additionally contoured hippocampi and SVZ. In order to maintain the constraints of conventionally contoured OARs, the beam directions had been left unchanged. The technique of intensity modulated stereotactic radiotherapy was already used in daily routine during study runtime. Our experiences resulted in a number of beamlets varying between 10 and 20. 14 beamlets were a satisfying approach for all cases used in this study. It was a goal to reduce the number of beamlets (7 beamlets) with the aim to get robust plans. As a positive side effect, these kind of plans provide shorter treatment times. In addition to that a better agreement between measured and calculated isodoses were expected, by avoiding very small beamlets. Therefore the first plan was generated using a leaf sequencer setup of 7 beamlets per beam; the second plan was generated using 14 beamlets per beam in order to optimize photon fluence profiles. For describing the results, these plans have been denominated “IMSRT-7” and “IMSRT-14” respectively. The constraints for the hippocampus and the SVZ were set at 6 Gy maximum total dose each. The maximum doses for hippocampi and SVZ obtained in the new plans were transformed into biologic effective doses (BED) using an alpha-beta ratio of 2 in the linear quadratic model.

The PTV was subject to an analysis of parameters describing the quality of dose distribution. The conformity index (CI) was defined as target volume (TV) divided by the volume receiving the reference dose (VRI) [9]. The homogeneity index (HI) was defined as maximum isodose in the target (Imax) divided by the reference isodose (RI). The quality of coverage (QoC) was defined as the minimal isodose encompassing the target (Imin) divided by reference isodose (RI).

**Figure 2:**
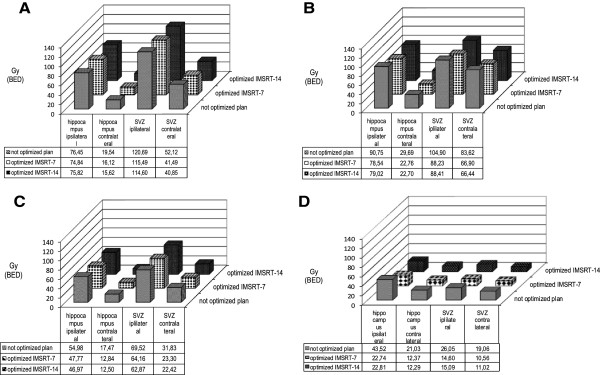
**Topographic type of 47 intracranial tumors: panels A –D modified according to**[10].

To explore the impact of topographic tumor site on quality indices and on potential hippocampus and SVZ sparing using IMSRT-7 and IMSRT-14 techniques, PTVs were assessed for their contact with the hippocampus and/or SVZ and the cortex as described elsewhere [10,11]. Four groups were segregated: group A (tumors contacting the SVZ and infiltrating cortex), group B (tumor contacting SVZ but not involving the cortex), group C (tumor involving cortex but not contacting the SVZ), group D (tumor involving neither SVZ nor cortex) (Figure 2).

#### Statistics

The software SPSS (version 18.0; SPSS, Chicago, IL, USA) was used to describe and analyse non-normal distributed data. Normal brain exposure and quality indices of the planning treatment volume, after different planning modes were tested using the non-parametric Wilcoxon-(rank sum) – test.

It was hypothesized that the following variables will impact the dose at the neurogenic stem cell niches: the structure to be spared (both hippocampi and SVZ), the topographic site of PTV (group A-D), its laterality (ipsilataral or contralateral to the PTV) and the mode of planning (not optimized, IMSRT-7, IMSRT-14). The agreement of biologic radiation doses for IMSRT-7 and IMSRT-14 plans at the hippocampi and the SVZ was visualized by Bland-Altman plot [12], which is a method to analyse the agreement of two different techniques by graphical presentation. For each structure-laterality-combination (Hippocampus/SVZ-ipsilateral/contralateral) of the two planning strategies (IMSRT-7; IMSRT-14), the individual differences of BED between IMSRT-14 and IMSRT-7 are plotted against their respective averages. The limits of agreement, which are given by mean value +/− 1.96 times the standard deviation of the differences, are computed also. These limits of agreement yield an area including 95% of the differences. Instead of using the original BED-data for the Bland-Altman plot the log-transformed BED-data were used, because a first inspection of the Bland-Altman plot with the original data showed systematic changes in the differences.

For this reason the BED data were also estimated in consideration of structure, group, laterality and treatment plan by applying linear mixed model using SAS 9.3. Thereby it is possible to consider and calculate the individual dependency concerning treatment plan, laterality and analyzed structures. The model for assessing the correlations between the different treatment techniques was based on unstructured covariance patterns. Concerning intraindividual factors of laterality and structure, no correlations are expected in terms of measured BEDs, which are also considered in the calculated model. In all analyses a p-value <0.05 was considered as statistically significant. To account for multiplicity we applied Bonferroni adjustment.

## Results

Demographic and tumor characteristics of 47 patients studied are given in Table 1. The tumor was located for 18 patients in the left hemisphere, for 18 patients in the right hemisphere and for 11 patients centrally. In these cases the tumor was arbitrarily allocated to the left or right hemisphere for analysis purposes according to its dominating laterality. To fulfil the conventional constraints, 27 patients received 3 D conformal plans and 20 patients initially m3-IMSRT in their original treatment plans without optimizing for hippocampi and SVZ. Dose constraints were respected in all conventionally contoured organs at risk in all plans. For optimized IMSRT-7 and IMSRT-14 plans the maximum doses for these OARs remained unchanged, while an optimization was performed for sparing of both hippocampi and SVZ. Topographic groups A, B, C and D comprised in 11, 8, 14 and 14 patients respectively (Table 1, Figure 2). None had multifocal tumor.

### Normal brain exposure, conformity index, homogeneity index and quality of coverage

The dose administered to 10%, 50% and 90% of the normal brain volume, outside the target volume (D 10, D 50, and D 90) varies for each treatment modality and proofed independent from the planning procedure (Table 2). However, when 90% of the normal brain volume is considered optimized, planning with IMSRT-7 or IMSRT-14 results in a small but significantly higher radiation exposure compared to the not optimized plans. D 90 slightly further increased using 14 beamlets compared to 7 (Table 2). Indeed exposure of a larger normal tissue volume at low dose levels is a hallmark of increasing number of beamlets in IMSRT. Both CI and HI of planning treatment volume show a small but significant improvement for the optimized IMSRT-7 and to the IMSRT-14 plans compared to the not optimized plans, albeit plans were not optimized for these parameters. The quality of coverage of the IMSRT plans compared to the not optimized plans remained unchanged (Table 2). Overall hippocampus and SVZ sparing was not achieved at the expense of quality of dose distribution at the target volume.

**Table 2:** Normal brain radiation exposure (median doses [Gy] and 25%- and 75%-quartiles) and conformity index, homogeneity index and quality of coverage of the planning target volume

	**Not optimized plan **^**a**^	**Optimized IMSRT-7**	**Optimized IMSRT-14**	**Not optimized vs. IMSRT-7 p**	**Not optimized vs. IMSRT-14 p**	**IMSRT 7 vs. IMSRT-14 p**
**Whole brain exposure**						
D 10 (Gy) ^b^	**25.290**	**26.240**	**24.450**	0.563	0.443	0.783
	Q25: 13.090	Q25:12.280	Q25: 12.400			
	Q75: 33.820	Q75: 34.740	Q75: 33.300			
D 50 (Gy) ^b^	**7.000**	**6.510**	**6.500**	0.589	0.663	0.829
	Q25: 3.960	Q25: 3.550	Q25: 3.480			
	Q75: 10.900	Q75: 10.080	Q75: 10.100			
D 90 (Gy) ^b^	**1.400**	**1.900**	**1.950**	<0.001	<0.001	0.423
	Q25: 1.100	Q25: 1.420	Q25: 1.460			
	Q75: 3.280	Q75: 3.300	Q75: 3.280			
conformality index ^c^	**0.980**	**0.990**	**1.000**	0.003	<0.001	0.005
	Q25: 0.970	Q25: 0.990	Q25: 0.990			
	Q75: 1.000	Q75: 1.000	Q75: 1.000			
homogeneity index ^c^	**1.140**	**1.110**	**1.090**	<0.001	<0.001	<0.001
	Q25: 1.100	Q25: 1.090	Q25: 1.070			
	Q75: 1.170	Q75: 1.120	Q75: 1.110			
quality of coverage ^c^	**0.920**	**0.930**	**0.920**	0.560	0.500	0.757
	Q25: 0.850	Q25: 0.890	Q25: 0.880			
	Q75: 0.950	Q75: 0.930	Q75: 0.940			

Comparison between not optimized dose distribution and plans optimized for hippocampus and SVZ sparing by intensity modulated stereotactic radiotherapy using 7 or 14 beamlets (IMSRT-7, IMSRT-14). p: p-value from non-parametric Wilcoxon-(rank sum)- test.

^a^ : 27 plans: 3 D conformal, 20 plans: IMSRT optimized for conventionally contoured organs at risk.

^b^ : D10, D50, D90: the median dose (Gy) + quartile to 10, 50 and 90% of the normal brain volume (ml) left after the PTV volume was excluded.

^c^ : conformity index: VRT/TV, homogeneity index: Imax/R, quality of coverage: Imin/RI.

### Itemized observation of parameters (laterality, plan, group, structure)

**Figure 3:**
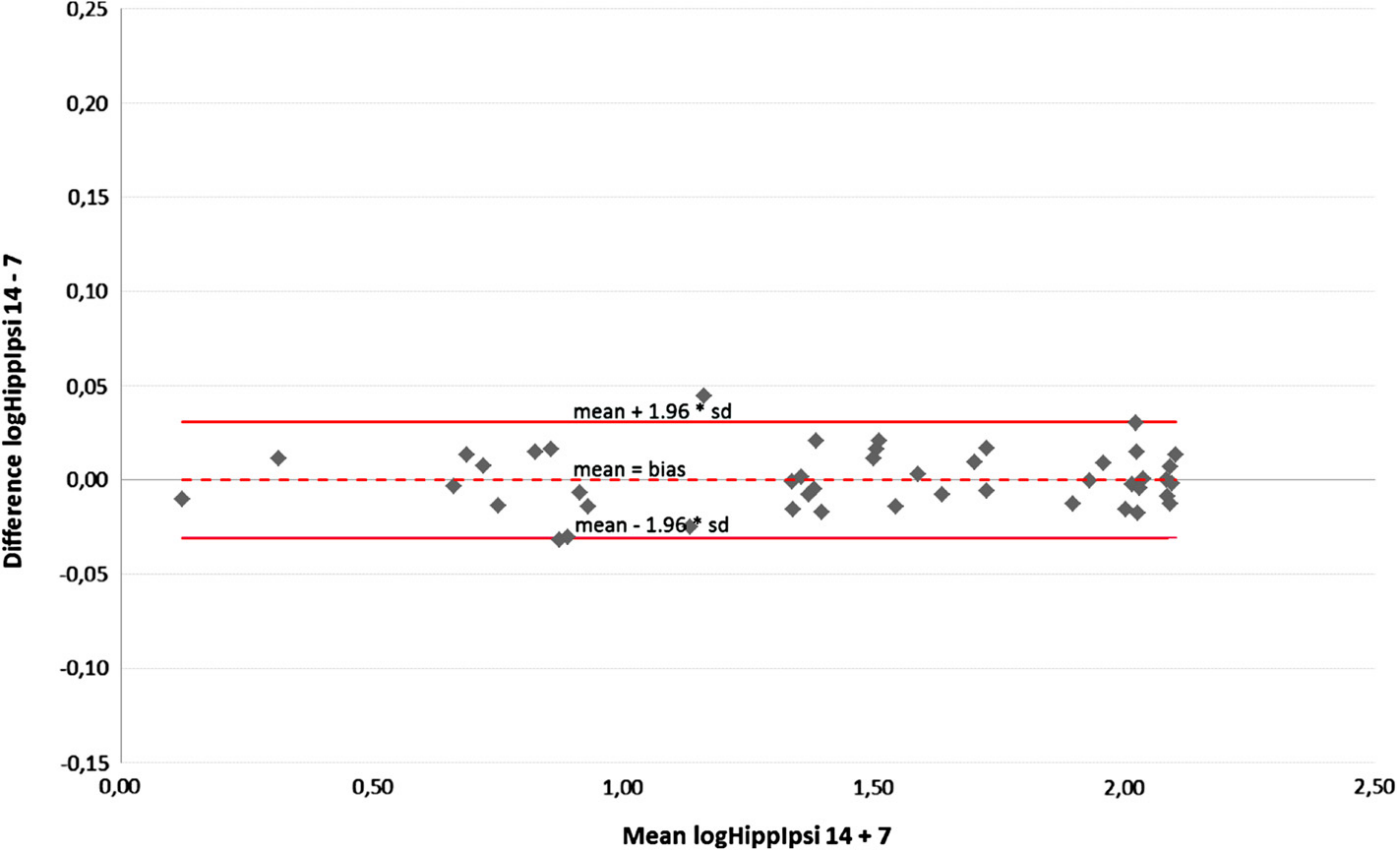
Mean biologic effective doses (BED Gy 2, linear quadratic model, alpha-beta ratio: 2) at the hippocampus and the subventricular zones ipsi- and contralateral for topographic groups A-D depending on the planning mode not optimized, IMRT-7 or IMRT-14 (definition see text).

For the entire group (n = 47) dose reduction was achieved by IMSRT 7 and IMSRT 14. Group D had noticeable lower BED doses compared with groups A-C overall. Achieved levels of sparing were favorable when the PTV was located contralateral to hippocampus and SVZ (Figure 3 A-D).

In line with the linear mixed model significances regarding variables structure to be spared (both hippocampi and SVZ), the topographic site of PTV (group A-D), its laterality (ipsilataral or contralateral to the PTV) and the mode of planning (not optimized, IMSRT-7, IMSRT-14) were observable (p < 0.0001), which could not be interpreted independent due to interactions (Table 3). No significances can be proved between laterality and structures as well as between laterality or structures and different treatment plans. In contrast laterality, structures and treatment plans interact with group classification significantly (p < 0.0001/ p = 0.0013/p = 0.0062). Due to inconclusiveness no additional higher-order interdependency could be established (p > 0.1237). A significant difference exists for the laterality concerning group A, B and C (p < 0.0001) and for the structures regarding to group A and B (p < 0.0001; p = 0.0028) on a closer examination of group assignment. Referring to treatment plans significant differences for group B, C and D are verifiable (p < 0.0001), but even more precisely for IMSRT 7 and IMSRT 14 in comparison with the not optimized treatment plans. Between IMSRT 7 and IMSRT 14 techniques no statistically significant difference is detected in each group.

**Table 3:** Tests of constant effects, p ≤ 0.005

**Effect**	**p-value**
laterality	<0.001
structure	<0.001
laterality*structure	0.39
plan	<0.001
laterality * plan	0.62
structure*plan	0.24
group	<0.001
laterality*group	<0.001
structure*group	0.00
plan*group	0.01

* is an indication for multi structure analysis (2 together).

Group assignment has an effect on laterality, structures and treatment plans, whereas the interaction between treatment plans and laterality, respectively structure is not significant. For group B, C and D is it possible to optimize the treatment plan concerning organs at risk with IMSRT 7 as well as IMSRT 14 compared to not optimized plans.

Comparison of 7 and 14 beamlets IMSRT concerning OARs

**Figure 4:**
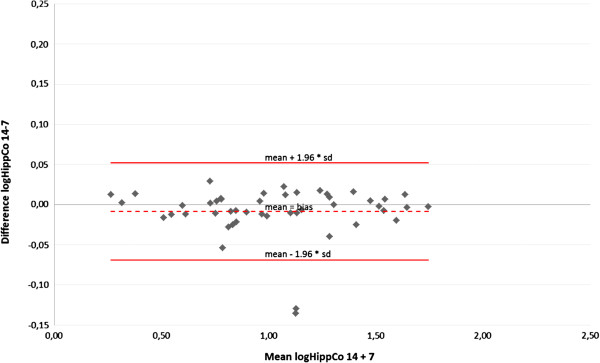
Mean and standard deviation of differences between total doses at the hippocampus ipsilateral to the PTV when irradiated with IMSRT-14 vs. IMSRT-7 in 46 patients (Bland-Altman plot); limits of agreement (mean+/-1.96*SD) in original scale: +8%/-7%.

**Figure 5:**
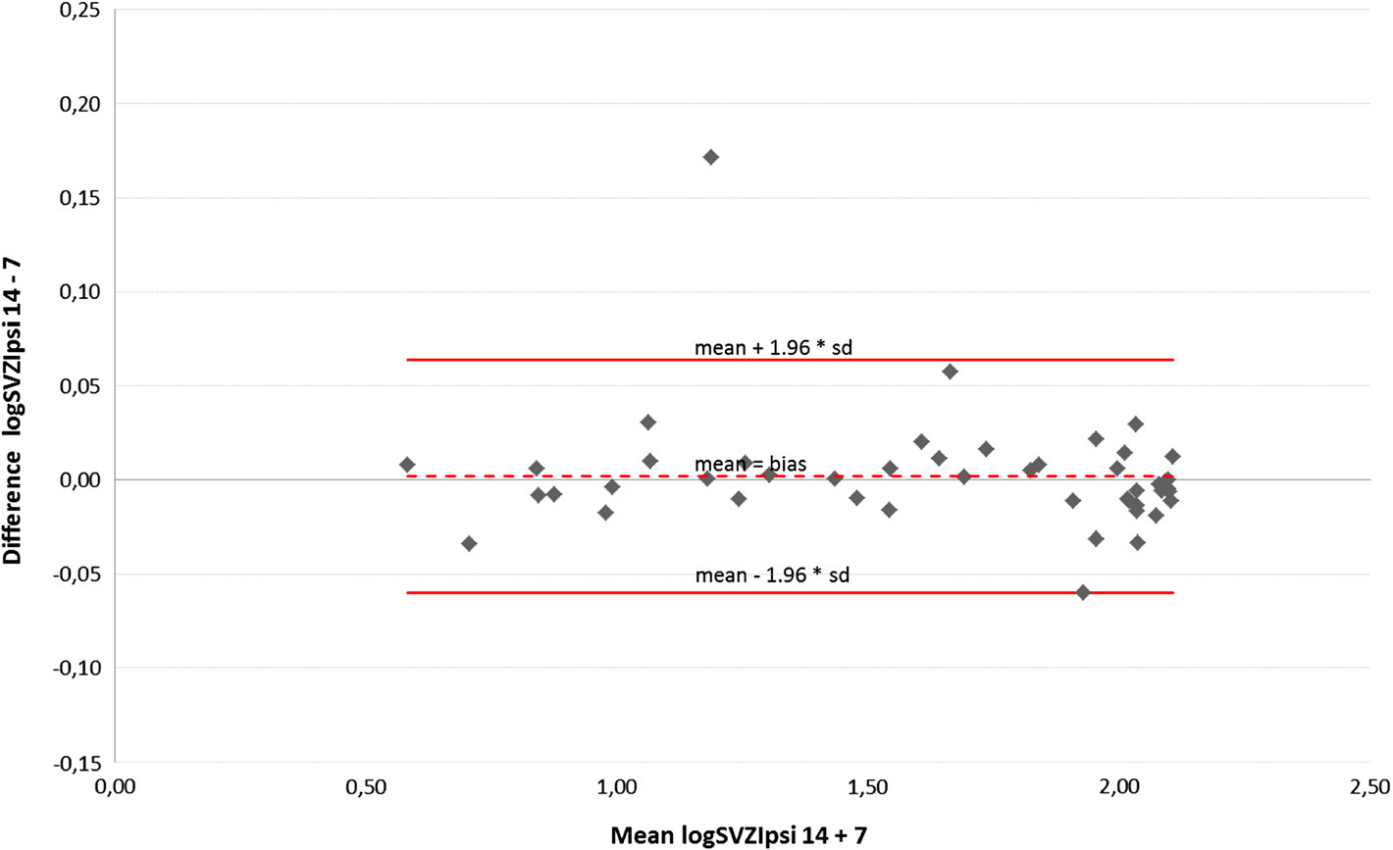
Mean and standard deviation of differences between total doses at the hippocampus contralateral to the PTV when irradiated with IMSRT-14 vs. IMSRT-7 in 47 patients (Bland-Altman plot); limits of agreement (mean+/-1.96*SD) in original scale: +13%/-15%.

**Figure 6:**
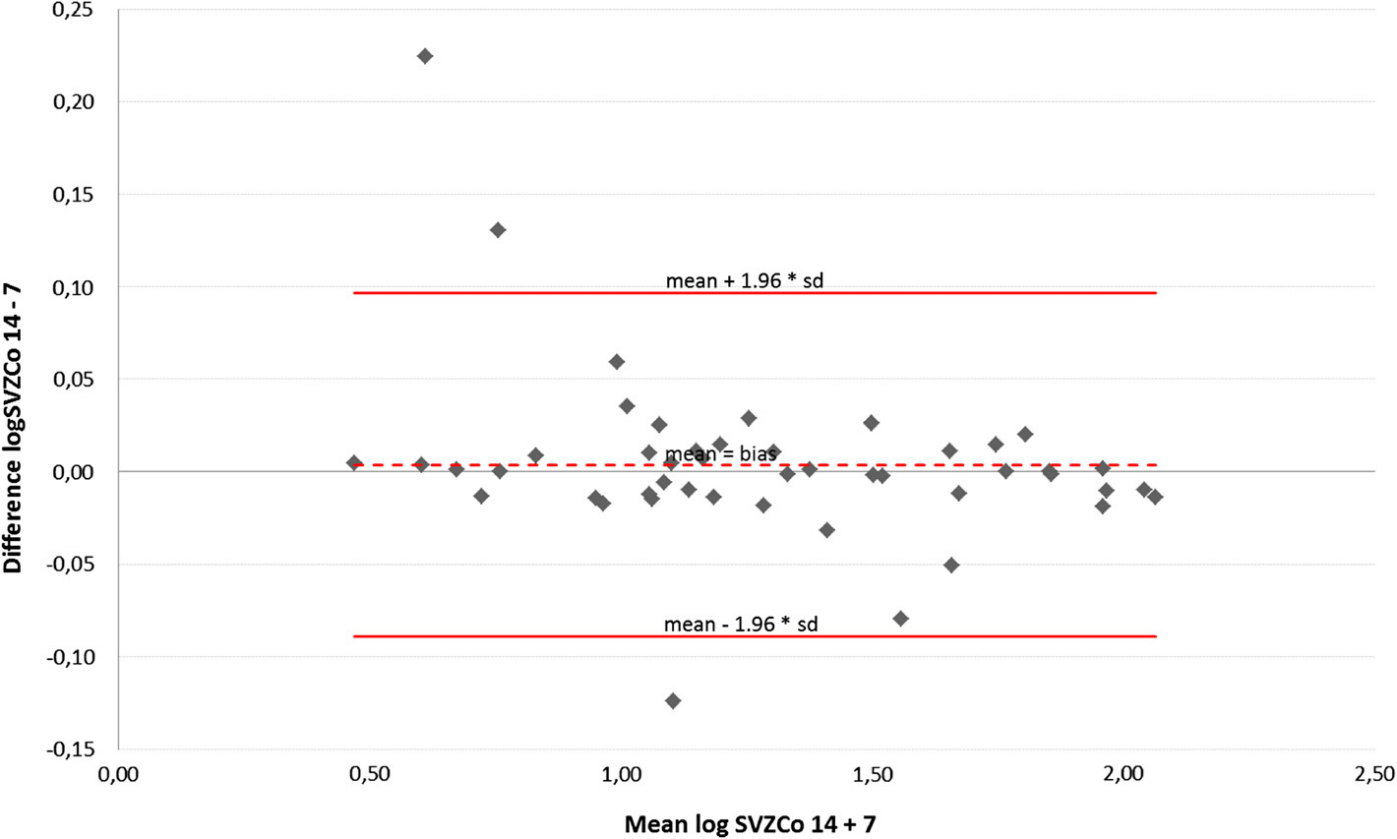
Mean and standard deviation of differences between total doses at the subventricular zone ipsilateral to the PTV when irradiated with IMSRT-14 vs. IMSRT-7 in 47 patients (Bland-Altman plot); limits of agreement (mean+/-1.96*SD) in original scale: +16%/-13%.

**Figure 7:**
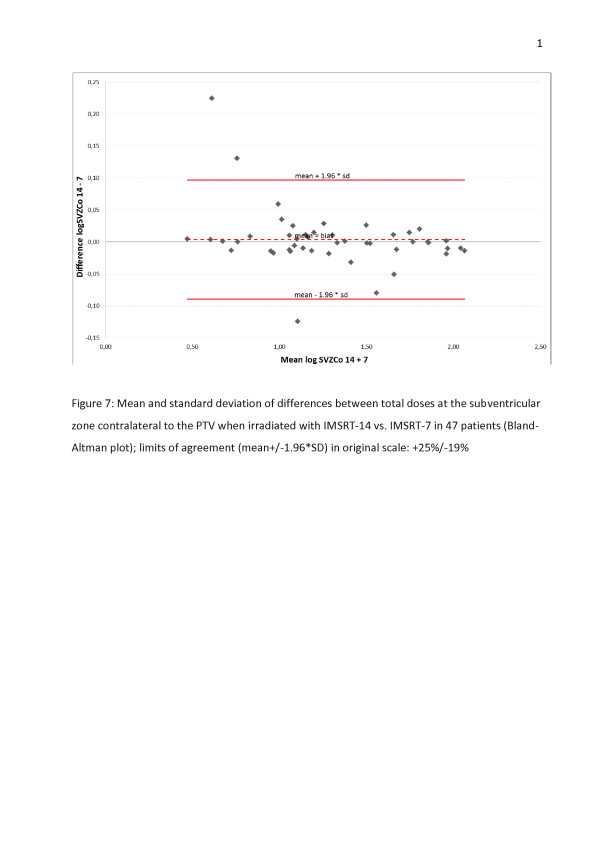
Mean and standard deviation of differences between total doses at the subventricular zone contralateral to the PTV when irradiated with IMSRT-14 vs. IMSRT-7 in 47 patients (Bland-Altman plot); limits of agreement (mean+/-1.96*SD) in original scale: +25%/-19%.

A Bland-Altman plot displays the agreement between der IMSRT-7 and IMRST-14 technique including the limits of agreement for each organ at risk (Figures 4, 5, 6, 7). A slight bias about the mean for ipsi- and contralateral hippocampus and SZV with marginal numbers of outliers beyond limits of agreement can be observed. The 14 beamlets IMSRT techniques provides total doses to contralateral hippocampus and SVZ, which ranges from 15% and 19% smaller, till 13% and 25% higher than with the 7 beamlets technique, respectively. The ipsilateral total doses for hippocampus and SVZ ranges from 93% to 108% and 87% to 116% with IMSRT-14 compared to IMRST-7. The total doses for the ipsilateral hippocampus is in 95% of all IMSRT-14 plans not more than 8% higher or 7% lower compared to IMSRT-7 plans. For the ipsilateral SVZ, 95% of all total dose figures for the IMSRT-14/IMSRT-7 ratio are within the range of 87% to 116%, i.e. it is expected that IMSRT-14 dose is not more than 13% lower or 16% higher compared to the IMSRT-7 dose. Furthermore, a small bias is inherent, when doses at contralateral hippocampus and SVZ are calculated using IMSRT-14. Techniques can be used interchangeable.

Adding them to the inverse optimization target function of a dedicated IMSRT considerably reduces the dose burdens at hippocampi and SVZ. Sparing at these structures critically depends on the relationship between the site of the tumor and the organ to be spared and its laterality. It seems reasonable to assume, that localization of tumor related to SVZ and hippocampus has an important impact on choice of treatment technique.

## Discussion

Neurotoxicity after radiotherapy has been classified as acute, subacute and late. Beside a variety of morphological changes detected in MRT (e.g. white matter lesions) and histologically, neurocognitive changes observed particularly in long term survivors have been associated with brain irradiation [6]. It is assumed, that the clinical counterpart of diffuse white matter changes may be a declined neurocognitive function, observed in children after fractionated total doses as low as 18 Gy to 24 Gy in 2 Gy fractions (BED = 36…48 Gy_2_ using α/β ratio of 2) [13]. This issue is of growing importance since survival has risen gradually over the past decade in virtually all malignant brain tumors. Recent reports on anaplastic glioma and GBM demonstrated an increased survival approaching a median of two years after escalated doses, e.g. applied by a stereotactic boost or by re-irradiation after recurrence, but also adverse long term neurotoxicity [14-17]. However, with more sensitive testing tools, neurocognitive decline can be detected as early as 4 months. After whole brain irradiation for brain metastases with 30 Gy, 52% of patients show a decline in learning and memory function compared to 24% in patients treated with stereotactic irradiation for 1–3 brain metastases alone [18]. Toxicity data from low-grade glioma patients treated with radiation doses of 50 Gy reported an actuarial 5-year incidence of mental decline of 5.3% [19]. Neurocognitive changes may be detected after a long clinically silent period up to 12 years after radiotherapy for anaplastic oligodendroglioma brain tumours [20], albeit cognitive dysfunction may be present before treatment begun [21]. Several factors are known to impact the development of neurocognitive disturbances; however a correlation between radiation dose and risk or severity of findings has not been established.

Extensive experimental research during the last 15 years has begun to elucidate the pathophysiologic mechanisms that may underlie neurocognitive dysfunction after radiotherapy of the brain. Radiotherapy and chemotherapy impact on normal (adult) neurogenesis originating in brain stem cells localized in dentate gyrus of the hippocampus and the subventricular zones around the lateral ventricles [6,22,23].

Hippocampal neurogenesis is a very complex physiological process [1]. Neuronal stem cells localized in the subventricular zone are considered to maintain white matter integrity. Generation of new neurons in the dentate gyrus of the hippocampus occurs in both rats and humans throughout life, and the addition of these new neurons is believed to be important to normal memory function [24-26]. Experimental and human studies have documented that radiation is able to disrupt the neurogenesis in the adult dentate gyrus even after exposure to single doses as low as 6 Gy, which is equivalent to 12 Gy given in 6 fractions with 2 Gy each (both resulting in BED of 24 Gy_2_ assuming a α/β ratio of 2) [4,23,27-30]. This may contribute to the clinical finding of neurocognitive decline after radiotherapy performed with low doses for brain metastases. Due to the proximity of the target volumes and their exposition with high doses while simultaneous sparing of hippocampal structures, the constraints for the latter may only be accomplished in a subgroup of tumor and / or using sophisticated and dedicated radiotherapy planning procedures. The present series demonstrate better sparing of contralateral neural stem cell niches compared to ipsilateral. The topographic site of PTV seems to impact the degree of sparing capability. In the present series, total doses at the neural stem cell niches are below doses associated with neurogenic impairment (after whole brain irradiation in children and adults) and even the dose range leading to a disruption of the neurogenesis in mouse models particularly in patients of group D (tumor involving neither subventricular zone nor cortex). Patients with such tumors may benefit most from dedicated IMSRT planning.

In conventional radiotherapy planning however neither the hippocampus nor the SVZ are contoured and considered as organs at risk during the topographical dose optimization process. Better sparing of normal brain tissue has been accomplished with IMSRT substituting 3D conformal dose escalated radiotherapy for high grade glioma with a consecutively reduced normal tissue complication probability in a radiobiological modelling study [31]. In patients requiring whole brain irradiation due to brain metastases, IMSRT has been shown to better spare the hippocampus both by linac based technique as well as by tomotherapy, when these organs at risk have been subjected to a high penalty in the inverse optimization algorithm [14,32,33].

IMSRT-7 and IMSRT-14 was optimized for sparing of the hippocampi and the SVZ. The dose homogeneity at the PTV as well as conformity index improved as an unscheduled side effect. The data presented demonstrate a considerable sparing achieved by contouring neuronal stem cell niches and exploiting the capabilities of micromultileaf collimation and dedicated dose optimization in a stereotactic treatment setting. The extent of sparing depends on the size of PTV but also its topographic site described by its relation to the cortex and the lateral ventricles. Additional modelling of high isodoses had only a marginal impact on sparing. This may be a valuable finding because it has the potential to prevent physicists from additional planning burden.

The Bland-Altman plot is a graphical method to show the relation between the differences and averages of two techniques, to control for systematic bias and to identify outliers. For clinically evaluation and interpretation of the data a back-transform of logarithm is practicable. The clinical importance of advancement between the two techniques can be measured against each other on the basis of percentages in original scale. The Bland-Altman plot also enables a statement relating to the comparison of IMSRT with 7 and 14 beamlets. Thereby no advantages with 14 beamlets over 7 beamlets are detectable.

The analysis seems reasonable to assume that localization of tumor related to SVZ and hippocampus has an important impact on choice of treatment technique. It is possible to optimize the treatment plan concerning organs at risk with IMSRT-7 as well as IMSRT-14 compared to non optimized plans for group B, C and D. On the contrary the different treatment techniques have no preserving effect for organs at risk when the tumor contacts SVZ and cortex irrespective of laterality.

The patient bases of this study were cases already treated with 3D-conformal SRT. The optimal beam directions for the 3D-SRT plans avoid direct interaction with any OAR and were already optimized by experienced medical physicists. The beam directions have been left unchanged because they allowed best sparing and were the ideal solution for every case. It is in our opinion highly recommended to use non-coplanar beam setups for cranial indications due to increase the number of degrees of freedom.

The aim of modern planning is not only to reduce the doses at the remaining normal brain and standard OAR, which has been correlated with different treatment methods [34], but also to spare sites where neurogenesis takes place, more specifically. However, an unsolved issue is the dose to be targeted at the hippocampus and the SVZ particularly according to the obviously multifactorial impairment of stem cell regeneration, in which corticoids and anticonvulsive drugs interfere with radiation [5]. In this series a total dose of 6 Gy over the entire series of fractionated radiotherapy was used as dose constraint. Others set the dose to zero, which seems unrealistic [8]. Recently a dose of 7 Gy at the hippocampus has been shown to impair late memory [35]. This figure could arbitrarily serve as a constraint in IMSRT however needs further confirmation.

## Competing interests

Thomas G. Wendt, Tilo Wiezorek and Nico Banz received honorarium from Brainlab AG independent from this work. All other authors declare that they have no competing interests.

## Authors’ contributions

JO carried out the contouring of critical structures, data management, performed the data analysis and statistics, reviewed the literature and contributed to writing the manuscript. TB prepared data set for contouring and generating experimental data. TGW was responsible for the study design, coordination, treatment planning and contributed to the final draft. MW advised statistical procedures, recalculated results and contributed to the draft. NB carried out MRI fusions and participated in the statistical analysis of the results and helped revising the draft. TW carried out the treatment planning and helped revising the draft. All authors read and approved the final manuscript.
